# Olmesartan/amlodipine/hydrochlorothiazide in participants with hypertension and diabetes, chronic kidney disease, or chronic cardiovascular disease: a subanalysis of the multicenter, randomized, double-blind, parallel-group TRINITY study

**DOI:** 10.1186/1475-2840-11-134

**Published:** 2012-10-30

**Authors:** Dean J Kereiakes, Steven G Chrysant, Joseph L Izzo, Thomas Littlejohn, Michael Melino, James Lee, Victor Fernandez, Reinilde Heyrman

**Affiliations:** 1 The Christ Hospital Heart and Vascular Center and The Carl and Edyth Lindner Center for Research and Education at the Christ Hospital, Cincinnati, OH, USA; 2 Oklahoma Cardiovascular and Hypertension Center and University of Oklahoma College of Medicine, Oklahoma City, OK, USA; 3 State University of New York at Buffalo, Buffalo, NY, USA; 4 Piedmont Medical Research Associates, Winston-Salem, NC, USA; 5 Daiichi Sankyo, Inc, Parsippany, NJ, USA; 6 Former employee, Daiichi Sankyo, Inc,, Parsippany, NJ, USA

**Keywords:** Hypertension, Diabetes mellitus, Chronic kidney disease, Cardiovascular diseases, Drug combinations

## Abstract

**Background:**

Patients with hypertension and cardiovascular disease (CVD), diabetes, or chronic kidney disease (CKD) usually require two or more antihypertensive agents to achieve blood pressure (BP) goals.

**Methods:**

The efficacy/safety of olmesartan (OM) 40 mg, amlodipine besylate (AML) 10 mg, and hydrochlorothiazide (HCTZ) 25 mg versus the component dual-combinations (OM 40/AML 10 mg, OM 40/HCTZ 25 mg, and AML 10/HCTZ 25 mg) was evaluated in participants with diabetes, CKD, or chronic CVD in the Triple Therapy with Olmesartan Medoxomil, Amlodipine, and Hydrochlorothiazide in Hypertensive Patients Study (TRINITY). The primary efficacy end point was least squares (LS) mean reduction from baseline in seated diastolic BP (SeDBP) at week 12. Secondary end points included LS mean reduction in SeSBP and proportion of participants achieving BP goal (<130/80 mm Hg) at week 12 (double-blind randomized period), and LS mean reduction in SeBP and BP goal achievement at week 52/early termination (open-label period).

**Results:**

At week 12, OM 40/AML 10/HCTZ 25 mg resulted in significantly greater SeBP reductions in participants with diabetes (−37.9/22.0 mm Hg vs −28.0/17.6 mm Hg for OM 40/AML 10 mg, −26.4/14.7 mm Hg for OM 40/HCTZ 25 mg, and −27.6/14.8 mm Hg for AML 10/HCTZ 25 mg), CKD (−44.3/25.5 mm Hg vs −39.5/23.8 mm Hg for OM 40/AML 10 mg, −25.3/17.0 mm Hg for OM 40/HCTZ 25 mg, and −33.4/20.6 mm Hg for AML 10/HCTZ 25 mg), and chronic CVD (−37.8/20.6 mm Hg vs −31.7/18.2 mm Hg for OM 40/AML 10 mg, −30.9/17.1 mm Hg for OM 40/HCTZ 25 mg, and −27.5/16.1 mm Hg for AML 10/HCTZ 25 mg) (*P*<0.05 for all subgroups vs dual-component treatments). BP goal achievement was greater for participants receiving triple-combination treatment compared with the dual-combination treatments, and was achieved in 41.1%, 55.0%, and 38.9% of participants with diabetes, CKD, and chronic CVD on OM 40/AML 10/HCTZ 25 mg, respectively. At week 52, there was sustained BP lowering with the OM/AML/HCTZ regimen. Overall, the triple combination was well tolerated.

**Conclusions:**

In patients with diabetes, CKD, or chronic CVD, short-term (12 weeks) and long-term treatment with OM 40/AML 10/HCTZ 25 mg was well tolerated, lowered BP more effectively, and enabled more participants to reach BP goal than the corresponding 2-component regimens.

**Trial Identification Number:**

NCT00649389

## Background

Hypertension is an important risk factor for development of cardiovascular disease (CVD) and chronic kidney disease (CKD) [1-3]. According to previous estimates, the National Health and Nutrition Examination Survey (2005–2008) showed that 98 million (21%) Americans have hypertension (defined as blood pressure [BP] >140/90 mm Hg). According to new estimates, an additional 52 million (11%) of American adults (for a total of 150 million adults [32%]) have uncontrolled BP requiring treatment (as defined by the American Heart Association Task Force as BP >140/90 mm Hg for low-risk individuals; >130/80 mm Hg for Framingham risk score >10%, CKD, diabetes, and CVD; and >120/80 for congestive heart failure). Adults with diabetes (50.6 million), CKD (43.7 million), and CVD (43.3 million) have the greatest prevalence of uncontrolled BP [4]. Hypertension is present in nearly 75% of patients with CVD, including coronary artery disease, stroke, diabetes, CKD, and peripheral artery disease [5-7]; however, it is estimated that only 53% of patients receiving antihypertensive treatment achieve BP control [7]. By 2030, it is estimated that 40.5% of the US population will have CVD [8].

The prevalence of hypertension is disproportionately high in patients who have diabetes [9], and individuals who have hypertension are nearly 2.5 times more likely to develop diabetes within 6 years than those without hypertension. Elevated BP is an important modifiable risk factor in patients with CKD, and BP reduction has the potential to both reduce cardiovascular death and attenuate progression of kidney disease [10-12]. It is estimated that triple-combination therapy is needed in at least 25% of all patients with hypertension in order to control BP [13]. Individuals who have hypertension and diabetes, CKD, or CVD are likely to need multiple antihypertensive agents to achieve the lower BP goals recommended in these high-risk populations [5,6]. Furthermore, single-pill combination therapy may result in increased adherence through reduction in pill burden and simplification of the therapeutic regimen [14]. A study including ~85,000 patients from Kaiser Permanente found that adherence decreased when the number of medications prescribed increased. In this study, antihypertensive medication adherence levels were 77.2%, 69.7%, 62.9%, and 55.5% in patients who received 1-, 2-, 3-, or 4-drug regimens, respectively [15].

The triple combination of olmesartan medoxomil (OM) 40 mg, amlodipine besylate (AML) 10 mg, and hydrochlorothiazide (HCTZ) 25 mg resulted in statistically significantly greater reductions in seated diastolic BP (SeDBP) and seated systolic BP (SeSBP) than the component dual-combination treatments in the Triple Therapy with Olmesartan Medoxomil, Amlodipine, and Hydrochlorothiazide in Hypertensive Patients Study (TRINITY) [16]. During the 40-week open-label extension period of the TRINITY study, continued administration of OM/AML/HCTZ triple-combination regimens demonstrated maintenance of the BP-lowering effects observed in the double-blind period of the study [17]. Furthermore, in the Blood Pressure Control in All Subgroups with Hypertension (BP-CRUSH) study (N=999), the addition of HCTZ to a single-pill combination of AML/OM allowed more patients to achieve SeBP goals [18].

The objective of these subgroup analyses was to compare the triple-combination treatment of OM 40/AML 10/HCTZ 25 mg with the component dual-combination treatments (OM 40/AML 10 mg, OM 40/HCTZ 25 mg, and AML 10/HCTZ 25 mg) in participants from the TRINITY study who had hypertension and diabetes (prespecified analysis) [19], CKD (prespecified analysis), or chronic CVD (post hoc analysis), and to evaluate the long-term efficacy and safety of OM/AML/HCTZ in these high-risk subgroups.

## Methods

### Study population

Individuals in the TRINITY study (NCT00649389; http://clinicaltrials.gov/ct2/show/NCT00649389) were aged ≥18 years with a mean SeBP ≥140/100 or ≥160/90 mm Hg (off antihypertensive medication) [16]. Persons with type 1 or type 2 diabetes controlled on a stable regimen with diet, insulin, or oral antidiabetes medications for ≥30 days and persons with CKD (creatinine clearance ≥30 mL/min and ≤60 mL/min) were eligible for inclusion. Persons with left ventricular hypertrophy, stable angina, peripheral vascular disease, and hypertensive retinopathy were also eligible for inclusion. Exclusion criteria included uncontrolled diabetes (with or without treatment) (ie, HbA1c >9.0%), stage IV CKD (ie, estimated glomerular filtration rate <30 mL/min/1.73 m^2^), history of a recent stroke or transient ischemic attack, myocardial infarction, percutaneous coronary intervention, coronary artery bypass surgery, and/or unstable angina within 6 months of enrollment or New York Heart Association class III or IV congestive heart failure. Persons with secondary hypertension, symptomatic resting bradycardia, heart block greater than first-degree atrioventricular block, and chronic atrial fibrillation or flutter were also excluded. The study was conducted in accordance with the institutional review board committee regulations and the Declaration of Helsinki [20]. All patients provided written informed consent at screening, before undergoing any study procedures.

### Study design

**Figure 1:**
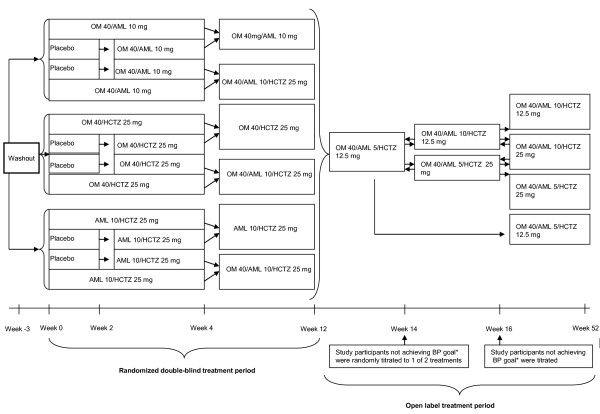
TRINITY study design (randomized, double-blind treatment period and open-label extension period). *BP goal is defined as <130/80 mg Hg for participants with diabetes, CKD, or chronic CVD. AML, amlodipine besylate; BP, blood pressure; CKD, chronic kidney disease; CVD, cardiovascular disease; HCTZ, hydrochlorothiazide; OM*,* olmesartan medoxomil. Adapted with permission from Elsevier [16].

TRINITY was a phase 3, randomized, parallel-group study conducted at 317 clinical sites in the United States and Puerto Rico and consisted of a 12-week double-blind treatment period followed by a 40-week open-label treatment period. Details of the 12-week study design have been published previously [16]; the study design for the 12-week double-blind and open-label treatment periods is summarized in Figure 1. Eligible study participants (stratified by age, race, and diabetes status) were randomized to a regimen that resulted in the 4 treatment groups that received treatment from weeks 4 to 12 (OM 40/AML 10 mg [single-pill combination], OM 40/HCTZ 25 mg [single-pill combination], AML 10/HCTZ 25 mg [not a single-pill combination; given separately], or OM 40/AML 10/HCTZ 25 mg [single-pill OM/HCTZ combination plus AML]). Although stratification for chronic CVD or CKD was not part of the stratification algorithm, there was a balanced distribution of participants within each of the treatment arms for both comorbidities in the total cohort. All participants received dual-combination treatment for 4 weeks, except for a subset of 36 control study participants who received placebo for 2 weeks and were subsequently switched to 1 of the 3 dual-combination treatments from week 2 to week 4. At week 4, participants were randomly maintained on dual-combination treatment to week 12 or given triple-combination treatment with OM 40/AML 10/HCTZ 25 mg until week 12. Participants completing the 12-week study were then enrolled in a 40-week open-label treatment period. All participants were switched to OM 40/AML 5/HCTZ 12.5 mg (administered as OM 40/AML 5 mg single-pill combination plus HCTZ 12.5 mg) at the start of the open-label extension period. Participants not achieving BP goal (<140/90 mm Hg or <130/80 mm Hg for participants with diabetes, CKD, or chronic CVD) after 2 weeks (week 14) were randomly titrated to 1 of 2 treatments (OM 40/AML 10/HCTZ 12.5 mg [administered as OM 40/AML 10 mg single-pill combination plus HCTZ 12.5 mg] or OM 40/AML 5/HCTZ 25 mg [administered as OM 40/AML 5 mg single-pill combination plus HCTZ 25 mg]). Participants not achieving BP goal 2 weeks after this titration (week 16) were further titrated to OM 40/AML 10/HCTZ 25 mg (administered as OM 40/AML 10 mg single-pill combination plus HCTZ 25 mg) [17]. 

### Study outcomes

The primary efficacy end point of the subgroup analyses was the least squares (LS) mean reduction from baseline in SeDBP at week 12 (primary efficacy registration requirement for OM/AML/HCTZ). Secondary end points included LS mean reduction in SeSBP and proportion of participants reaching BP goal (<130/80 mm Hg) at week 12. For the open-label extension period, efficacy endpoints included mean SeBP and proportion of participants achieving BP goal (<130/80 mm Hg) at weeks 12, 14, 16, and 52/early termination (ET). Safety assessments included adverse events (AEs), clinical laboratory tests, vital signs, physical examinations, and 12-lead electrocardiograms.

### Statistical analysis

The primary efficacy analysis population was defined as all participants who received at least 1 dose of study medication and had assessments for SeDBP at baseline and at least once post dose. The safety population (for AE assessment) included all participants receiving at least 1 dose of study medication at or beyond the week 4 visit of the 12-week double-blind period.

For the double-blind treatment period, two-sided *P* values for testing the significance of the triple-combination treatment versus each dual-combination treatment were derived from an analysis of covariance (ANCOVA) model that had baseline BP as a covariate and fixed effects of final randomized treatment, subgroup (eg, diabetes status subgroup), and final randomized treatment by subgroup interaction. For each comparison, the LS mean difference, corresponding standard error (SE), and two-sided *P* values were derived from the model. The proportion of participants reaching BP goal in each treatment group was summarized and analyzed using the chi square test. Comparisons between triple-combination treatment and each dual-combination treatment were performed using Fisher’s exact test at a 0.05 significance level. The last observation carried forward (LOCF) approach was used for ET measurements during double-blind treatment. Summary statistics by dosing regimen were used to describe SeDBP and SeSBP at each open-label visit week and the proportion of participants reaching BP goal.

## Results

### Study participant disposition

**Figure 2:**
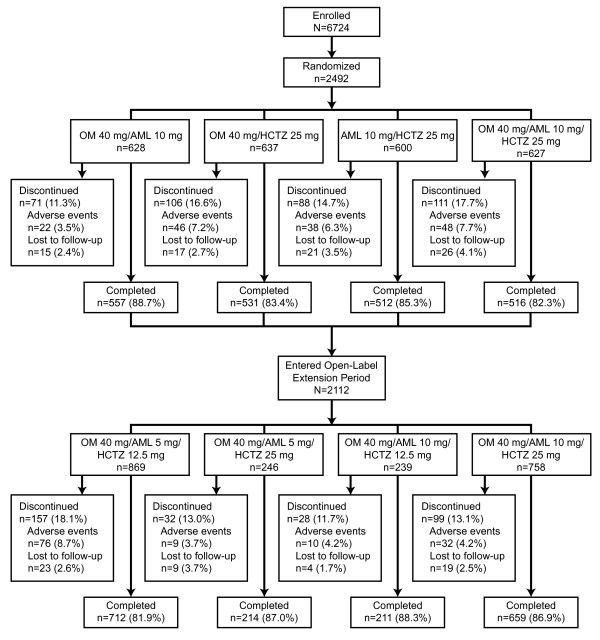
**Participant disposition. **AML*,* amlodipine besylate; HCTZ*,* hydrochlorothiazide; OM*,* olmesartan medoxomil.

A total of 2492 participants were randomized into the study, of whom 2116 completed the 12-week double-blind treatment period, 2112 entered the 40-week open- label extension period (4 participants completed the double-blind period of the study, but did not continue in the open-label period: 2 individuals withdrew consent and 2 individuals discontinued from the study due to AEs), and 1796 completed the study (Figure 2). Of the randomized participants, 387 (15.5%) had diabetes, 103 (4.1%) had CKD, and 227 (9.1%) had chronic CVD. Participants with comorbid diabetes, CKD, and/or CVD were included in each applicable subgroup. Baseline characteristics of these subgroups are summarized by treatment arm in Table 1. For the diabetes, CKD, and chronic CVD subgroups, mean age was 58.7, 70.1, and 59.6 years and baseline BP was 171.7/98.5, 173.3/96.6, and 173.0/100.4 mm Hg, respectively. 

**Table 1:** Demographic and baseline characteristics of the diabetes, CKD, and chronic CVD subgroups (randomized set)

	**Diabetes (n=387)**				**CKD (n=103)**				**Chronic CVD (n=227)**			
	**OM 40/ AML 10 mg**	**OM 40/ HCTZ 25 mg**	**AML 10/ HCTZ 25 mg**	**OM 40/ AML 10/ HCTZ 25 mg**	**OM 40/ AML 10 mg**	**OM 40/ HCTZ 25 mg**	**AML 10/ HCTZ 25 mg**	**OM 40/ AML 10/ HCTZ 25 mg**	**OM 40/ AML 10 mg**	**OM 40/ HCTZ 25 mg**	**AML 10/ HCTZ 25 mg**	**OM 40/ AML 10/ HCTZ 25 mg**
	**(n=100)**	**(n=99)**	**(n=92)**	**(n=96)**	**(n=29)**	**(n=25)**	**(n=29)**	**(n=20)**	**(n=56)**	**(n=61)**	**(n=55)**	**(n=55)**
Age, mean (SD), yrs	59.7 (8.9)	60.3 (8.7)	56.5 (9.4)	58.1 (9.4)	71.0 (7.8)	71.5 (9.4)	69.4 (9.5)	68.1 (10.0)	60.2 (11.2)	61.1 (9.8)	58.2 (11.0)	58.6 (10.9)
Male, n (%)	60 (60.0)	54 (54.5)	52 (56.5)	51 (53.1)	9 (31.0)	10 (40.0)	10 (34.5)	5 (25.0)	40 (71.4)	38 (62.3)	34 (61.8)	39 (70.9)
Ethnicity												
Hispanic/Latino, n (%)	21 (21.0)	18 (18.2)	13 (14.1)	15 (15.6)	1 (3.4)	2 (8.0)	3 (10.3)	2 (10.0)	8 (14.3)	5 (8.2)	7 (12.7)	8 (14.5)
Race												
White, n (%)	72 (72.0)	73 (73.7)	58 (63.0)	67 (69.8)	18 (62.1)	19 (76.0)	21 (72.4)	13 (65.0)	40 (71.4)	45 (73.8)	37 (67.3)	35 (63.6)
Black, n (%)	26 (26.0)	24 (24.2)	29 (31.5)	25 (26.0)	9 (31.0)	5 (20.0)	7 (24.1)	6 (30.0)	16 (28.6)	16 (26.2)	18 (32.7)	17 (30.9)
Obesity, n (%)*	77 (77.0)	74 (74.7)	71 (77.2)	73 (76.0)	9 (31.0)	2 (8.0)	7 (24.1)	6 (30.0)	32 (57.1)	40 (65.6)	35 (63.6)	33 (60.0)
Hypertension duration, mean (SD), y	12.8 (9.6)	13.2 (10.5)	11.2 (9.6)	10.7 (9.8)	12.1 (8.0)	14.0 (10.0)	13.0 (9.3)	14.8 (14.4)	12.0 (8.0)	15.0 (12.3)	12.9 (9.9)	13.3 (11.0)
Baseline BP, mean (SD), mm Hg	172.2/98.6 (13.5/7.5)	173.1/97.8 (14.7/7.6)	170.7/99.3 (13.6/6.7)	170.7/98.3 (16.0/6.9)	174.4/96.4 (12.5/8.8)	172.2/96.0 (11.6/7.6)	172.9/97.4 (17.1/6.6)	173.8/96.6 (16.5/6.5)	173.4/101.0 (16.2/10.6)	172.8/99.3 (11.7/9.3)	174.2/100.8 (16.7/7.1)	171.7/100.4 (11.0/9.1)

*BMI ≥30 kg/m^2^.

*AML*, amlodipine; *BMI*, body mass index; *CKD*, chronic kidney disease; *CVD*, cardiovascular disease; *HCTZ*, hydrochlorothiazide; *OM*, olmesartan medoxomil; *SD*, standard deviation.

### Efficacy

#### Week 12

**Figure 3:**
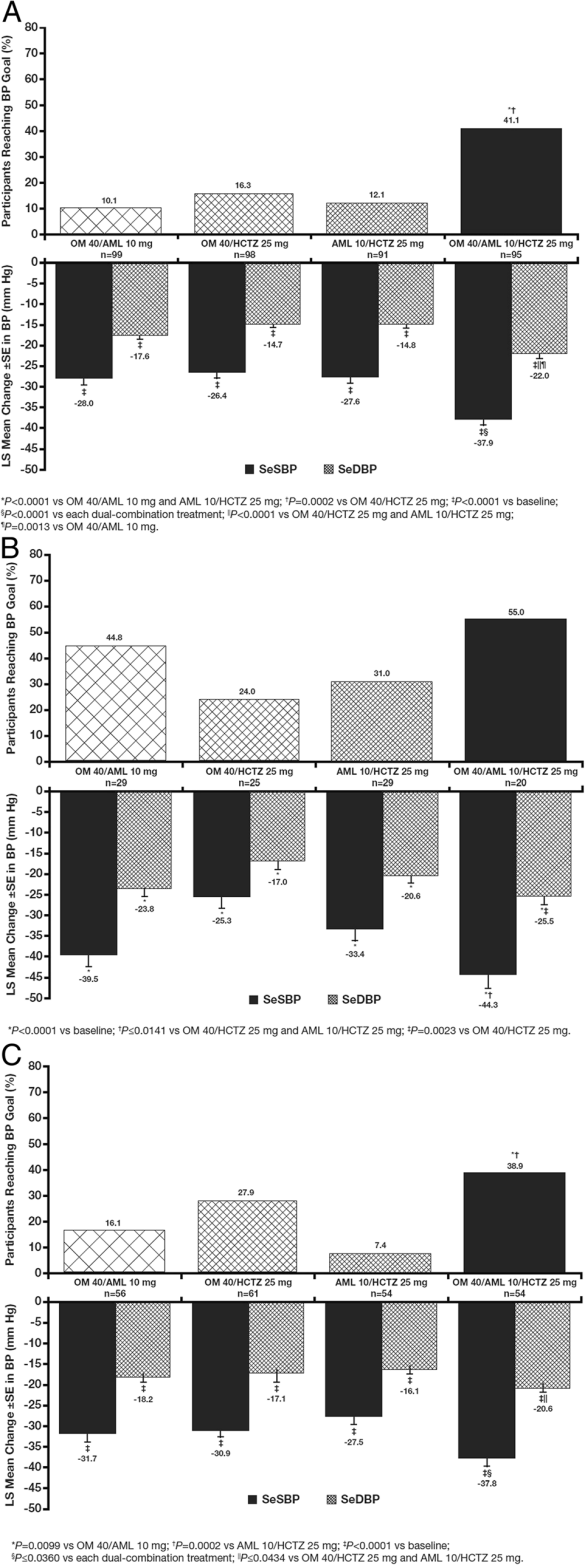
**Diabetes (A), CKD (B), and chronic CVD (C) subgroups: LS Mean (SE) reductions in SeBP and proportion of participants reaching BP goal (<130/80 mm Hg) at week 12 (LOCF). **Specific *P* values are found beneath each panel. AML, amlodipine besylate; BP, blood pressure; CKD, chronic kidney disease; CVD, cardiovascular disease; HCTZ, hydrochlorothiazide; LOCF, last observation carried forward; LS, least squares; OM, olmesartan medoxomil; SE, standard error.

In study participants with diabetes, CKD, and chronic CVD, triple-combination treatment with OM 40/AML 10/HCTZ 25 mg resulted in greater BP reductions and a greater proportion of participants achieving BP goal of <130/80 mm Hg at week 12 (LOCF) compared with the dual-combination treatments (Figure 3A, 3B and 3C). Mean SeBP at week 12 was 131.8/77.3, 127.0/72.9, and 132.3/79.9 mm Hg for participants with diabetes, CKD, and chronic CVD, respectively. The proportion of participants receiving triple-combination treatment who reached BP goal in each of these subgroups was 41.1% (diabetes), 55.0% (CKD), and 38.9% (chronic CVD). 

#### Week 52

Mean BP and proportion of participants reaching BP goal (<130/80 mm Hg) during the open-label extension period in the diabetes, CKD, and chronic CVD subgroups is summarized in Table 2. In the diabetes, CKD, and chronic CVD subgroups, mean SeBP at week 52/ET ranged from 121.4–136.7/74.9–79.7, 118.4–134.0/70.9–75.0, and 127.1–138.4/77.1–82.3 mm Hg, respectively. BP goal was reached by an average of 44.3%, 51.7%, and 33.7% of participants with diabetes, CKD, and chronic CVD, respectively. 

**Table 2:** Mean BP and proportion of participants reaching BP goal (<130/80 mm Hg) at weeks 12 and 52/ET

**Time point**	**Diabetes**	**CKD**	**Chronic CVD**
**Week 12**			
**n**	333	93	191
OM 40/AML 5/HCTZ 12.5 mg*	138.9/81.2 (17.4/9.6)	135.0/76.5 (20.6/10.9)	137.7/81.4 (15.7/10.3)
% to goal	22	40	25
**Week 52/ET**			
**n**	62	29	56
OM 40/AML 5/HCTZ 12.5 mg	121.4/74.9 (14.5/8.8)	118.4/70.9 (12.6/7.9)	127.1/77.1 (18.8/12.4)
% to goal	65	79	54
n	34	13	29
OM 40/AML 5/HCTZ 25 mg	129.5/77.2 (15.6/9.9)	126.5/72.4 (22.5/10.5)	134.2/78.0 (17.0/11.6)
% to goal	38	54	35
n	40	14	20
OM 40/AML 10/HCTZ 12.5 mg	128.8/77.0 (11.6/8.5)	122.3/70.9 (15.6/8.1)	132.9/77.6 (11.1/8.5)
% to goal	50	43	20
n	197	36	86
OM 40/AML 10/HCTZ 25 mg	136.7/79.7 (15.2/9.7)	134.0/75.0 (17.7/10.3)	138.4/82.3 (16.2/10.6)
% to goal	24	31	27

BP data are mean (SD), mm Hg.

*Week 12 data are from the end of the double-blind treatment period before all participants were switched to OM 40/AML 5/HCTZ 12.5 mg at the beginning of the open-label extension period.

*AML*, amlodipine; *BP*, blood pressure; *CKD*, chronic kidney disease; *CVD*, cardiovascular disease; *ET*, early termination; *HCTZ*, hydrochlorothiazide; *OM*, olmesartan medoxomil; *SD*, standard deviation.

### Safety

No new safety concerns were identified for either the triple- or dual-combination treatments that were not known to occur with the individual component therapies. Treatment-emergent AEs (TEAEs) for the diabetes, CKD, and chronic CVD subgroups during the double-blind treatment period are summarized in Table 3. At week 12, 205 (56.3%), 56 (57.7%), and 119 (57.8%) study participants with diabetes, CKD, and chronic CVD, respectively, had a TEAE. Across all treatment groups for each subgroup, most TEAEs were considered mild or moderate in severity. In total, 11 (3.0%), 3 (3.1%), and 6 (2.9%) participants with diabetes, CKD, and chronic CVD had a serious AE (SAE) and 11 (3.0%), 3 (3.1%), and 6 (2.9%) discontinued from the study due to a TEAE. 

**Table 3:** Study participants with treatment-emergent adverse events at week 12

	**Diabetes**				**CKD**				**Chronic CVD**			
**All data n (%)**	**OM 40/ AML 10 mg**	**OM 40/ HCTZ 25 mg**	**AML 10/ HCTZ 25 mg**	**OM 40/ AML 10/ HCTZ 25 mg**	**OM 40/ AML 10 mg**	**OM 40/ HCTZ 25 mg**	**AML 10/ HCTZ 25 mg**	**OM 40/ AML 10/ HCTZ 25 mg**	**OM 40/ AML 10 mg**	**OM 40/ HCTZ 25 mg**	**AML 10/ HCTZ 25 mg**	**OM 40/ AML 10/ HCTZ 25 mg**
	**(n=96)**	**(n=91)**	**(n=85)**	**(n=92)**	**(n=29)**	**(n=22)**	**(n=28)**	**(n=18)**	**(n=53)**	**(n=54)**	**(n=50)**	**(n=49)**
All TEAEs*	47 (49.0)	49 (53.8)	51 (60.0)	58 (63.0)	16 (55.2)	11 (50.0)	20 (71.4)	9 (50.0)	23 (43.4)	32 (59.3)	30 (60.0)	34 (69.4)
Severe TEAEs	4 (4.2)	4 (4.4)	5 (5.9)	6 (6.5)	0	2 (9.1)	2 (7.1)	1 (5.6)	2 (3.8)	2 (3.7)	2 (4.0)	5 (10.2)
**Drug-related TEAEs**^†^	21 (21.9)	16 (17.6)	17 (20.0)	25 (27.2)	5 (17.2)	8 (36.4)	10 (35.7)	3 (16.7)	9 (17.0)	11 (20.4)	13 (26.0)	17 (34.7)
**Discontinuations**												
TEAEs	2 (2.1)	2 (2.2)	2 (2.4)	5 (5.4)	0	1 (4.5)	2 (7.1)	0	0	3 (5.6)	0	3 (6.1)
Drug-related TEAEs^†^	1 (1.0)	0	0	5 (5.4)	0	0	1 (3.6)	0	0	0	0	2 (4.1)
**TEAEs (>5% in any treatment group)**^‡^												
Dizziness	3 (3.1)	3 (3.3)	3 (3.5)	7 (7.6)	1 (3.4)	1 (4.5)	3 (10.7)	1 (5.6)	1 (1.9)	5 (9.3)	2 (4.0)	7 (14.3)
Headache	5 (5.2)	8 (8.8)	2 (2.4)	5 (5.4)	1 (3.4)	3 (13.6)	1 (3.6)	0	1 (1.9)	3 (5.6)	1 (2.0)	2 (4.1)
Urinary tract infection	3 (3.1)	3 (3.3)	3 (3.5)	6 (6.5)	0	0	0	3 (16.7)	0	0	1 (2.0)	2 (4.1)
Upper respiratory tract infection	2 (2.1)	2 (2.2)	2 (2.4)	2 (2.2)	3 (10.3)	0	1 (3.6)	2 (11.1)	0	0	1 (2.0)	0
Bronchitis	2 (2.1)	0	1 (1.2)	3 (3.3)	0	1 (4.5)	0	2 (11.1)	0	3 (5.6)	2 (4.0)	1 (2.0)
Edema, peripheral	9 (9.4)	1 (1.1)	4 (4.7)	9 (9.8)	1 (3.4)	1 (4.5)	5 (17.9)	1 (5.6)	3 (5.7)	1 (1.9)	4 (8.0)	4 (8.2)
Fatigue	5 (5.2)	10 (11.0)	6 (7.1)	2 (2.2)	0	1 (4.5)	2 (7.1)	0	2 (3.8)	5 (9.3)	1 (2.0)	3 (6.1)
Joint swelling	1 (1.0)	0	2 (2.4)	1 (1.1)	4 (13.8)	0	0	1 (5.6)	1 (1.9)	1 (1.9)	3 (6.0)	3 (6.1)
Muscle spasms	4 (4.2)	2 (2.2)	0	3 (3.3)	1 (3.4)	2 (9.1)	0	0	0	3 (5.6)	3 (6.0)	0
Nausea	0	3 (3.3)	2 (2.4)	4 (4.3)	1 (3.4)	0	2 (7.1)	0	0	2 (3.7)	0	4 (8.2)
Diarrhea	1 (1.0)	2 (2.2)	2 (2.4)	2 (2.2)	4 (13.8)	0	1 (3.6)	0	2 (3.8)	0	1 (2.0)	0
Constipation	0	0	0	5 (5.4)	0	1 (4.5)	1 (3.6)	0	1 (1.9)	0	0	2 (4.1)
Hypokalemia	0	0	2 (2.4)	1 (1.1)	0	0	4 (14.3)	0	0	2 (3.7)	3 (6.0)	0

*TEAEs were AEs that emerged during treatment (absent pre-treatment or worsened relative to pre-treatment). TEAEs are defined as having a start date on/after the first dose of double-blind study medication and up to the first dose of open-label study medication for participants continuing into the open-label period; or, for early terminated participants, up to and including 14 days after the last dose date of double-blind study medication. All TEAEs are counted under the treatment the participant received from week 4 to week 12.

^†^Drug-related was defined as definitely, probably, or possibly related to randomized study medication.

^‡^TEAEs presented occurred in >5% and at least 3 study participants in any treatment group.

*AE*, adverse event; *AML*, amlodipine besylate; *CVD*, cardiovascular disease; *CKD*, chronic kidney disease; *HCTZ*, hydrochlorothiazide; *OM*, olmesartan medoxomil; *TEAE*, treatment-emergent adverse event.

The safety profile for these 3 subgroups during the open-label treatment period is summarized in Table 4. The most common TEAEs (≥5%) across doses during the open-label extension period were upper respiratory tract infection (11; 5.4%) in participants with diabetes; dizziness (3; 9.4%), nasopharyngitis (3; 7.7%), and urinary tract infection (3; 7.7%) in participants with CKD; and dizziness (6; 6.3%) and cough (5; 5.3%) in participants with chronic CVD. 

**Table 4:** Study participants with adverse events during the open-label treatment period by onset dosing regimen

	**Diabetes**				**CKD**				**Chronic CVD**			
**All data n (%)**	**OM 40/ AML 5/ HCTZ 12.5 mg**	**OM 40/ AML 5/ HCTZ 25 mg**	**OM 40/ AML 10/ HCTZ 12.5 mg**	**OM 40/ AML 10/ HCTZ 25 mg**	**OM 40/ AML 5/ HCTZ 12.5**	**OM 40/ AML 5/ HCTZ 25 mg**	**OM 40/ AML 10/ HCTZ 12.5 mg**	**OM 40/ AML 10/ HCTZ 25 mg**	**OM 40/ AML 5/ HCTZ 12.5**	**OM 40/ AML 5/ HCTZ 25 mg**	**OM 40/ AML 10/ HCTZ 12.5 mg**	**OM 40/ AML 10/ HCTZ 25 mg**
	**(n=334)**	**(n=139)**	**(n=143)**	**(n=203)**	**(n=93)**	**(n=35)**	**(n=32)**	**(n=39)**	**(n=191)**	**(n=74)**	**(n=70)**	**(n=95)**
All AEs*	135 (40.4)	40 (28.8)	48 (33.6)	120 (59.1)	43 (46.2)	13 (37.1)	15 (46.9)	23 (59.0)	88 (46.1)	25 (33.8)	20 (28.6)	54 (56.8)
Severe AEs	12 (3.6)	2 (1.4)	4 (2.8)	8 (3.9)	4 (4.3)	1 (2.9)	1 (3.1)	4 (10.3)	7 (3.7)	1 (1.4)	3 (4.3)	5 (5.3)
**Drug-related AEs**^†^	37 (11.1)	9 (6.5)	12 (8.4)	28 (13.8)	14 (15.1)	4 (11.4)	5 (15.6)	8 (20.5)	30 (15.7)	8 (10.8)	4 (5.7)	24 (25.3)
**Discontinuations**												
AEs	5 (1.5)	2 (1.4)	1 (0.7)	9 (4.4)	8 (8.6)	1 (2.9)	2 (6.3)	3 (7.7)	9 (4.7)	1 (1.4)	0	6 (6.3)
AEs starting in open-label extension period	5 (1.5)	2 (1.4)	1 (0.7)	8 (3.9)	7 (7.5)	1 (2.9)	2 (6.3)	3 (7.7)	9 (4.7)	1 (1.4)	0	6 (6.3)
Drug-related AEs^†^	3 (0.9)	2 (1.4)	1 (0.7)	3 (1.5)	6 (6.5)	0	2 (6.3)	2 (5.1)	6 (3.1)	0	0	3 (3.2)
**AEs (>5% in any treatment group)**^‡^												
Dizziness	10 (3.0)	4 (2.9)	3 (2.1)	10 (4.9)	6 (6.5)	0	3 (9.4)	2 (5.1)	7 (3.7)	0	2 (2.9)	6 (6.3)
Urinary tract infection	13 (3.9)	4 (2.9)	2 (1.4)	9 (4.4)	1 (1.1)	1 (2.9)	0	3 (7.7)	4 (2.1)	0	1 (1.4)	3 (3.2)
Upper respiratory tract infection	8 (2.4)	0	6 (4.2)	11 (5.4)	5 (5.4)	0	2 (6.3)	2 (5.1)	3 (1.6)	1 (1.4)	3 (4.3)	3 (3.2)
Nasopharyngitis	2 (0.6)	3 (2.2)	2 (1.4)	8 (3.9)	2 (2.2)	1 (2.9)	1 (3.1)	3 (7.7)	6 (3.1)	1 (1.4)	0	3 (3.2)
Cough	6 (1.8)	4 (2.9)	3 (2.1)	4 (2.0)	1 (1.1)	1 (2.9)	2 (6.3)	2 (5.1)	0	1 (1.4)	2 (2.9)	5 (5.3)

*Adverse events starting before the open-label extension period and not resolved by week 12 were counted under the final dosing regimen.

^†^Drug-related was defined as definitely, probably, or possibly related to randomized study medication.

^‡^AEs presented occurred in >5% and at least 3 study participants in any treatment group. Although a participant may have had 2 or more AEs, the participant is counted only once within a category. The same participant may appear in different categories.

*AML*, amlodipine besylate; *CVD*, cardiovascular disease; *CKD*, chronic kidney disease; *HCTZ*, hydrochlorothiazide; *OM*, olmesartan medoxomil; *TEAE*, treatment-emergent adverse event.

Although small changes were observed in serum chemistry and hematology parameters, there was no apparent relationship to the dose or combination of therapy, and none were considered clinically significant. For the total cohort at week 12/ET, mean glucose levels (non-fasted) were 113.6 mg/dL (baseline: 108.5 mg/dL) for OM 40/AML 10/HCTZ 25 mg; 114.1 mg/dL (baseline: 110.2 mg/dL) for OM 40/AML 10 mg; 113.3 mg/dL (baseline: 109.9 mg/dL) for OM 40/HCTZ 25 mg; and 117.0 mg/dL (baseline: 107.7 mg/dL) for AML 10/HCTZ 25 mg. Mean creatinine at week 12/ET was 0.98 mg/dL (baseline: 0.92 mg/dL) for OM 40/AML 10/HCTZ 25 mg; 0.92 mg/dL (baseline: 0.95 mg/dL) for OM 40/AML 10 mg; 1.0 mg/dL (baseline: 0.94 mg/dL) for OM 40/HCTZ 25 mg; and 0.94 mg/dL (baseline: 0.93 mg/dL) for AML 10/HCTZ 25 mg. Creatinine clearance was 112.5 mL/min (baseline: 119.5 mL/min); 121.8 mL/min (baseline: 121.8 mL/min); 109.5 mL/min (baseline: 117.7 mL/min); and 118.6 mL/min (baseline: 120.5 mL/min) for the respective treatment groups. No effects on heart rate, electrocardiograms, or physical examinations were observed during the treatment period of the study.

## Discussion

The current subgroup analyses demonstrated the effectiveness of triple-combination treatment in difficult-to-treat participants with hypertension and diabetes, CKD, or chronic CVD. BP reductions with triple-combination treatment in these subgroups at week 12 were comparable to those for the overall study cohort (−37/22 mm Hg) [16]. Long-term treatment with varying doses of OM/AML/HCTZ in these subgroups resulted in similar BP-lowering effects for those receiving triple-combination treatment during the double-blind period of the study. Triple-combination treatment also enabled these high-risk subgroups to achieve BP goal (<130/80 mm Hg) at week 12 and at week 52. Triple-combination treatment was well tolerated in these subgroups, with a low percentage of participants discontinuing treatment across treatment groups and no clinically meaningful differences in AEs. The presence of diabetes, CKD, or chronic CVD did not change the AE profile of the drugs used in this analysis from that observed in the total TRINITY study cohort [16].

It has been estimated that at least 75% of patients with hypertension require combination therapy to maintain BP control [14], and large clinical trials have reported that 23-54% of participants require 3 or more antihypertensive agents [21-24]. Thus, there is a growing emphasis on the need for practical strategies to consistently achieve and maintain BP goals with the use of multiple antihypertensive agents in clinical practice [14].

Single-pill combination therapy may improve BP control through regimen simplification, which may lead to an improvement in patient adherence, reduction in physician visits, and attainment of BP goals [14,16,25-30]. Single-pill combinations have the potential to increase adherence to therapy compared with free-dose combinations [31,32], particularly in patients with hypertension and cardiovascular comorbidities. Such patients are at increased risk of nonadherence due to the need for multiple medications for a variety of comorbidities (eg, dyslipidemia, diabetes) in addition to antihypertensive medications [33,34].

Combining antihypertensive agents from different classes has been estimated to produce BP reductions approximately 5 times greater than doubling the dose of any single agent [35]. A meta-analysis has shown that one drug at standard dose compared with a combination of 3 drugs at half the standard dose reduced the incidence of coronary heart disease by about 24% and 45%, respectively, and stroke by 33% and 60%, respectively, in individuals aged 60 to 69 years with SeBP of 150/90 mm Hg [36]. The complementary mechanisms of action of an angiotensin receptor blocker, calcium channel blocker, and diuretic result in each agent targeting a separate pathway and provide coverage for multiple different pathways contributing to hypertension [14,25,35]. Additionally, in patients with evidence of renal disease or at greater risk of developing renal disease, such as those with diabetes mellitus, it is recommended to use renin-angiotensin-aldosterone system blocker–based combination therapy [37,38].

Guidelines recognize that multiple antihypertensive agents are often required in patients with diabetes and CKD [5]. In addition, the American Heart Association recommends starting with ≥2 antihypertensive agents in patients with coronary artery disease [6]. The Seventh Report of the Joint National Committee on Prevention, Detection, Evaluation, and Treatment of High Blood Pressure (JNC 7) recommends a lower BP goal (<130/80 mm Hg) for patients with diabetes or CKD, as both are major risk factors for CVD [5]. Similarly, the American Heart Association/American College of Cardiology recommends a BP goal of <130/80 mm Hg for patients at high risk for or with demonstrated coronary artery disease [6]. The American College of Cardiology Foundation/American Heart Association/American Medical Association–Physician Consortium for Performance Improvement 2011 Performance Measures for Adults with Coronary Artery Disease and Hypertension report also states that lower BP targets may be appropriate for some patients with coronary artery disease [39].

Despite the evidence supporting the benefit of targeted BP control in high-risk patient groups, some investigators have raised questions regarding incremental benefit with more aggressive BP goals [40-42]. The European Society of Hypertension guidelines note that many recommendations on hypertension management are based on post hoc analyses rather than prospective randomized trial data, with the latter being a preferable evidence base for BP target recommendations in different patient groups [43]. At present, it appears reasonable to adhere to currently established guideline targets and goals of <130/80 mm Hg in these high-risk patient groups.

Limitations to the current subgroup and post hoc analyses include that statistical analyses between the subgroups were not completed because of the unequal participant numbers in the subgroups. In addition, the TRINITY study only evaluated the highest doses of each of the 3 agents in the dual- and triple-combination regimens; therefore, it does not provide information on the efficacy and safety of the lower dosing regimens. This study also excluded individuals with illnesses such as symptomatic heart failure and therefore it cannot be determined whether the triple-combination treatment is appropriate for this patient population; caution must be exercised regarding generalizability of these data to the overall population.

## Conclusions

Treatment with OM 40/AML 10/HCTZ 25 mg resulted in greater reductions in SeBP compared with each dual-combination treatment in high-risk subgroups of participants (diabetes, CKD or chronic CVD) from the TRINITY study. Also, a greater proportion of participants in each subgroup achieved BP goal (<130/80 mm Hg) with the triple combination at week 12 compared with the dual-combination treatments, which was maintained during the open-label period of the study. Long-term triple-combination therapy was well tolerated.

## Abbreviations

AEs: Adverse events; AML: Amlodipine besylate; ANCOVA: Analysis of covariance; BP: Blood pressure; CKD: Chronic kidney disease; CVD: Cardiovascular disease; ET: Early termination; HCTZ: Hydrochlorothiazide; JNC 7: Seventh report of the joint national committee on prevention, detection, evaluation, and treatment of high blood pressure; LOCF: Last observation carried forward; LS: Least squares; OM: Olmesartan medoxomil; SAE: Serious AE; SE: Standard error; SeDBP: Seated diastolic blood pressure; SeSBP: Seated systolic blood pressure; TEAEs: Treatment-emergent AEs; TRINITY: Triple therapy with olmesartan medoxomil, amlodipine, and hydrochlorothiazide in hypertensive patients study.

## Competing interests

Dean J. Kereiakes, MD reports no disclosure information. Steven G. Chrysant, MD, has served as a consultant and speakers bureau member for and received grant/research support and honoraria from Daiichi Sankyo Inc. Joseph L. Izzo, Jr., MD, has served as a consultant or investigator for Daiichi Sankyo Inc, Boehringer-Ingelheim, Novartis, GlaxoSmithKline, Takeda Pharmaceuticals, and Forest Laboratories. Michael Melino, PhD, James Lee, PhD, and Victor Fernandez, BS, are employees of Daiichi Sankyo, Inc. Reinilde Heyrman, MD, is a former employee of Daiichi Sankyo, Inc.

## Authors’ contributions

The trial was designed by Daiichi Sankyo, Inc. in conjunction with the investigators. All authors contributed to the study design; data analysis/interpretation; drafting, critical revision, and approval of the manuscript. Medpace, Inc. (Cincinnati, Ohio), a contract research organization, performed project management, data management, clinical and safety monitoring, and statistical analyses in conjunction with Daiichi Sankyo, Inc. The authors are saddened to report the passing in March 2011 of Thomas Littlejohn, III, MD, esteemed physician, investigator, colleague, and co-author of posters and publications from the TRINITY study. His contributions to this manuscript were invaluable. The authors are saddened to report the passing in March 2011 of Thomas Littlejohn, III, MD, esteemed physician, investigator, colleague, and co-author of posters and publications from the TRINITY study. His contributions to this manuscript were invaluable.
